# Characterization of education, technical practices and attitudes of Portuguese pharmacy technicians towards manipulation of cytotoxic drugs

**DOI:** 10.1177/10781552231190025

**Published:** 2023-07-25

**Authors:** Daniela Campos, Inês Silva, Mariana Rego, Patrícia Correia, Fernando Moreira

**Affiliations:** 1 203255 Escola Superior de Saúde, Instituto Politécnico do Porto, Rua Dr António Bernardino de Almeida, Porto, Portugal; 2 Centro de Investigação em Saúde e Ambiente 203255 , Escola Superior de Saúde, Instituto Politécnico do Porto, Rua Dr António Bernardino de Almeida, Porto, Portugal

**Keywords:** Cytotoxic, chemotherapy, antineoplastic agents, occupational exposure, pharmacy technicians

## Abstract

Pharmacy professionals that manipulate cytotoxic drugs need to undergo educational programs, adopt the most convenient practices, and use appropriate equipment to avoid, as far as possible, occupational exposure to cytotoxic drugs. The main goal of this work is to characterize the education, technical practices, and attitudes towards cytotoxic drugs, of Portuguese pharmacy technicians. A questionnaire comprising eleven questions deemed pertinent was elaborated and subsequently validated by a pilot test. The anonymous, web-based survey was conducted between December 2022 and January 2023, by graduated pharmacy technicians that had manipulated cytotoxic drugs between 2017 and 2022. A total of 77 pharmacy technicians responded to the survey. Although sixty-six pharmacy technicians (86%) had been trained before beginning to manipulate cytotoxic drugs, the promotion of regular post-admission training by the institutions is sparse – only assumed by 53% of the pharmacy technicians (*n* = 41). All participants reported using gloves and gown during manipulation and the use of double gloves was common (99%; *n* = 76). Compliances with the recommended limit time for uninterrupted manipulation activity (82%; *n* = 63) and systematic double-checking (86%; *n* = 66) were high, but the regular use of sterile gauze around syringe connection sites 58% (*n* = 45), was less frequent. None of the surveyed pharmacy technicians used closed-system transfer devices (CSTD) and 41 (53%) of those who used spikes did not thoughtfully use these devices. The implementation of regular training programs in manipulating cytotoxic drugs should be fostered, to promote the more judicious use of engineering controls and transversal adoption of the safest technical practices.

## Introduction

In 2020, the worldwide incidence of cancer exceeded 19 million new cases and the absolute prevalence surpassed 50 million cases. By 2040, the incidence is projected to increase to 30 million new cases, thus accentuating the trend of increasing cancer patients.^1^ Despite the emergence of new approaches for cancer treatment, such as biological molecules and immune-mediated therapies, chemotherapy remains the most widely used treatment.^2^ It is foreseeable that more and more qualified professionals in pharmacy will be needed to ensure the treatment of cancer patients, through the preparation of solutions containing cytotoxic drugs.

In the 1970s, the possibility of serious occupational risks in healthcare professionals working with antineoplastic drugs began to be admitted.^3,4^ Since then, several studies have already demonstrated serious adverse effects of mutagenic nature, reproductive toxic effects, and secondary cancer among healthcare workers exposed to cytotoxic drugs.^5–9^ Even if exposed to trace amounts, the exposure of professionals is repetitive, thus resulting in cumulative toxicity with potentially serious consequences.^10^ Several studies have used the analysis of urinary samples from professionals to identify and quantify the cytotoxic agents present in this biological marker of exposure. Some studies reported the presence of several cytotoxic drugs in urine samples from exposed professionals, such as cyclophosphamide in pharmacists,^11,12^ pharmacy technicians^11–14^ and nurses;^11,12,15^ methotrexate, ifosfamide, carboplatin, and cisplatin in pharmacy technicians;^14^ and 5-fluorouracil, paclitaxel and anthracyclines in pharmacy technicians.^13^ On the other hand, there are also studies reporting minimal or no urinary levels of cyclophosphamide and ifosfamide in samples from pharmacy technicians, pharmacists and nurses.^16^ The observed differences, despite using similar sampling and analytical methodologies, suggest that there is indeed variability in exposure to cytotoxic agents in different work settings. Disparities in the handling technique of cytotoxic drugs and differences in the use of personnel protective equipment (PPE) are among the main reasons for variability.^11^

Since the contamination of professionals working with cytotoxic drugs can be affected by the manipulation technique and the compliance with the correct use of PPE, the adequate training of the staff involved in the process of handling drugs is of the greatest importance.^17^ Particularly among nurses, prior studies described the training programs that professionals who handled cytotoxics had attended as well as their compliance with guidelines regarding several aspects (use of PPE, use of biological safety cabinets (BSC), etc.).^10,18–30^ Nevertheless, the description of the practices and attitudes of pharmacy technicians in this regard has been less common.

The main goal of this work is to characterize the education, technical practices, and attitudes towards cytotoxic drugs, adopted by Portuguese pharmacy technicians that manipulate cytotoxic drugs, and to conclude on the fulfilment of the recommendations provided by guidelines.

## Methods

To perform this observational cross-sectional study, a questionnaire was elaborated to be responded to by pharmacy technicians.

Given the main objectives of this research, the questionnaire conceptual framework was developed considering the dependent variable “Training on Cytotoxic Drugs Manipulation and Adopted Procedures by Pharmacy Technicians” as potentially affected by the independent variables (i) training prior admission in cytotoxic drugs manipulation; (ii) periodical education and evaluation promoted by the employer; (iii) use of PPE; (iv) practices and attitudes towards cytotoxic drugs manipulation; (v) use of engineering controls; (vi) major concerns and difficulties of pharmacy technicians that manipulate cytotoxics.

To ensure that any questions beyond the scope of the research were excluded, the questionnaire was reviewed by two experts in the field of cytotoxic drug handling concerning the relevance of all questions. Eleven questions were deemed relevant. Providing succinct questionnaires is associated with increased participants’ adherence.^31^

Then, the questionnaire was validated by a pilot test that aimed to verify if the questions were correctly formulated and written in a comprehensive manner for the target population. Accordingly, the pilot test was answered by eleven pharmacy technicians. By asking the potential respondents to answer the questionnaire, it was possible to clarify dubious questions, include available options for each question as exhaustive as possible and detect flaws in the questionnaire in terms of content, grammar, and format.

The questionnaire mainly included close-ended questions, since, for most questions, the range of answers was well-known, and the options were limited. Still, an open-ended question was included to allow the respondents to express their opinions freely, regarding the major concerns and difficulties that pharmacy technicians experience in handling cytotoxics. The answers were grouped into categories, according to their similarity.

In questions that involved assessing habits, Likert scales were considered, since it is acknowledged that it provides a more reliable measure of strength for a particular attitude or belief than ambiguous terms alone.^31^

The anonymous, web-based survey was conducted between December 10, 2022, and January 31, 2023. The questionnaire was prepared on the Google Forms platform and was disseminated on social network pages of pharmacy professional groups, ensuring free and expressly voluntary access to all the public that they reach.

Handling cytotoxic drugs between 2017 and 2022; working in Portuguese Hospital Units; being a graduated pharmacy technician; and voluntarily responding to the questionnaire; were considered inclusion criteria.

Given the predicted restricted number of eligible respondents, the questionnaire had no personnel or demographic questions. Although demographic characterization of the study population could be interesting, it was decided to forego this data at the expense of guaranteeing the anonymity of the participants.

The relevance of most of the questions included in the present survey is corroborated by previous studies in which the use of PPE, use of BSC, frequency of educational programs, and use of puncturing devices alternatives to needles were addressed to evaluate the adherence to safe handling practices of cytotoxic drugs.^18,21,27,29,32^

Data were analysed using Microsoft Excel, version 365, enabling the presentation of simple frequencies and prevalences.

The study was approved by the Ethics Committee of the Escola Superior de Saúde do Instituto Politécnico do Porto (Document approved no. CE0093C).

## Results

The few existent studies that characterized the practices and education of pharmacy technicians surveyed 24 professionals from Canada^32,33^ and 183 pharmacy practitioners (sum of pharmacy technicians and pharmaceutics; the number of pharmacy technicians was not revealed) from the USA.^34^ In the present study, a total of 77 pharmacy technicians responded to the survey, featuring the training, practices, and attitudes towards cytotoxic drugs, in a European country (Portugal).

### Training in the manipulation of cytotoxic drugs

In the present study, a total of 66 pharmacy technicians (86%) had performed training before starting to manipulate cytotoxic drugs. The remaining 14% (*n* = 11) admitted having started manipulating cytotoxics without prior training. In a study of 253 nurses involved in the preparation of cytotoxic drugs, 29.6% of the professionals had received training before admittance.^19^ Similar results were observed in a study in which 22.2% of the pharmacists surveyed (*n* = 27), had received training before handling cytotoxic drugs.^20^ There are also health professionals involved in the preparation of cytotoxic drugs who revealed that they had not received any prior training.^35^ In institutions where training and assessment are not carried out, health professionals involved in the handling of cytotoxic drugs assume that training before admission should always be mandatory, being considered one of the main justifications that limit the safety of handling those agents.^10^ Although the performance of prior training is superior in this survey, an increasing tendency to train newly recruited professionals to handle cytotoxics should be aimed, to reach the desirable 100%, in future surveys. According to several guidelines, such as ISOPP,^36^ OSHA,^37^ and ESOP,^38^ the training of pharmacy professionals that manipulate cytotoxic drugs before admission is mandatory.

**Figure 1.**
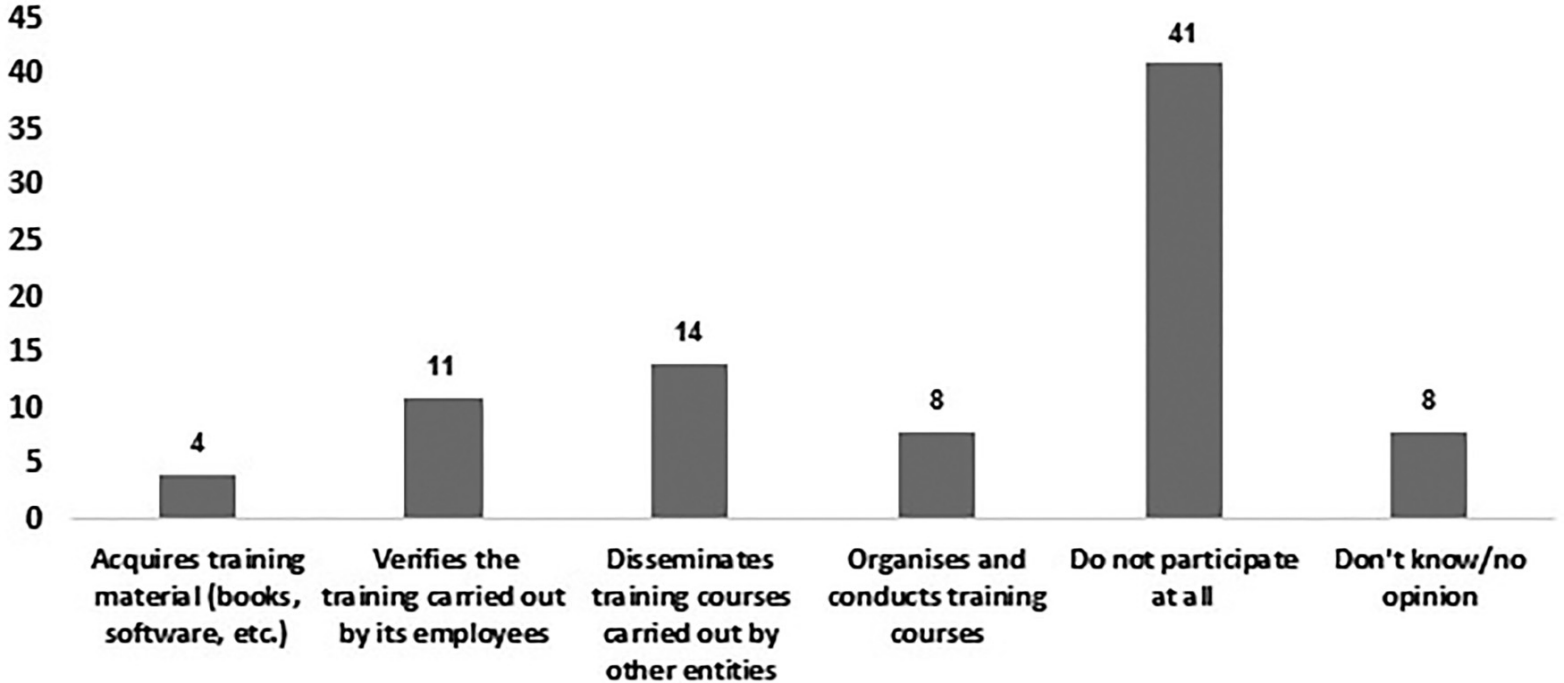
Type of participation of the employing institutions of pharmacy technicians (*n* = 77) in specific training in the handling of cytotoxic drugs, after the professional had started to perform this task.

The training and assessment of professionals dedicated to the manipulation of cytotoxic agents should also be performed regularly, after admission. According to the ISOPP guidelines, the training should be repeated, at least, every 2–3 years and assessment should be performed yearly.^36^ Several other guidelines corroborate the need for regular training and assessment.^37,39–41^ Still, the involvement of employing institutions in providing regular training for professionals handling cytotoxic drugs has often been portrayed as scarce or non-existent.^10,21,35^ The results observed in the present study regarding the participation of the employing institutions confirm that in the studied population, there is also little investment in regular training and assessment of pharmacy technicians after their enrolment in handling cytotoxic drugs. Forty-one participants (53%) stated that the employer had no participation in their training at any level and only eleven (14%) recognized that the employer enquires them about performed training. The main contribution of the employers in regular training of the professionals is the dissemination of training courses carried out by third parties (*n* = 14, 18%) (Figure 1). This might be worrisome since lack of training was already pointed out by professionals as the main cause of the spills and accidents that occurred during cytotoxic handling.^19^ Using linear regression models, it was concluded that differences in urinary concentrations of cyclophosphamide in exposed professionals were associated in a statistically significant manner with the absence of cytotoxic handling training.^11^

### Personnel protective equipment (PPE)

**Figure 2.**
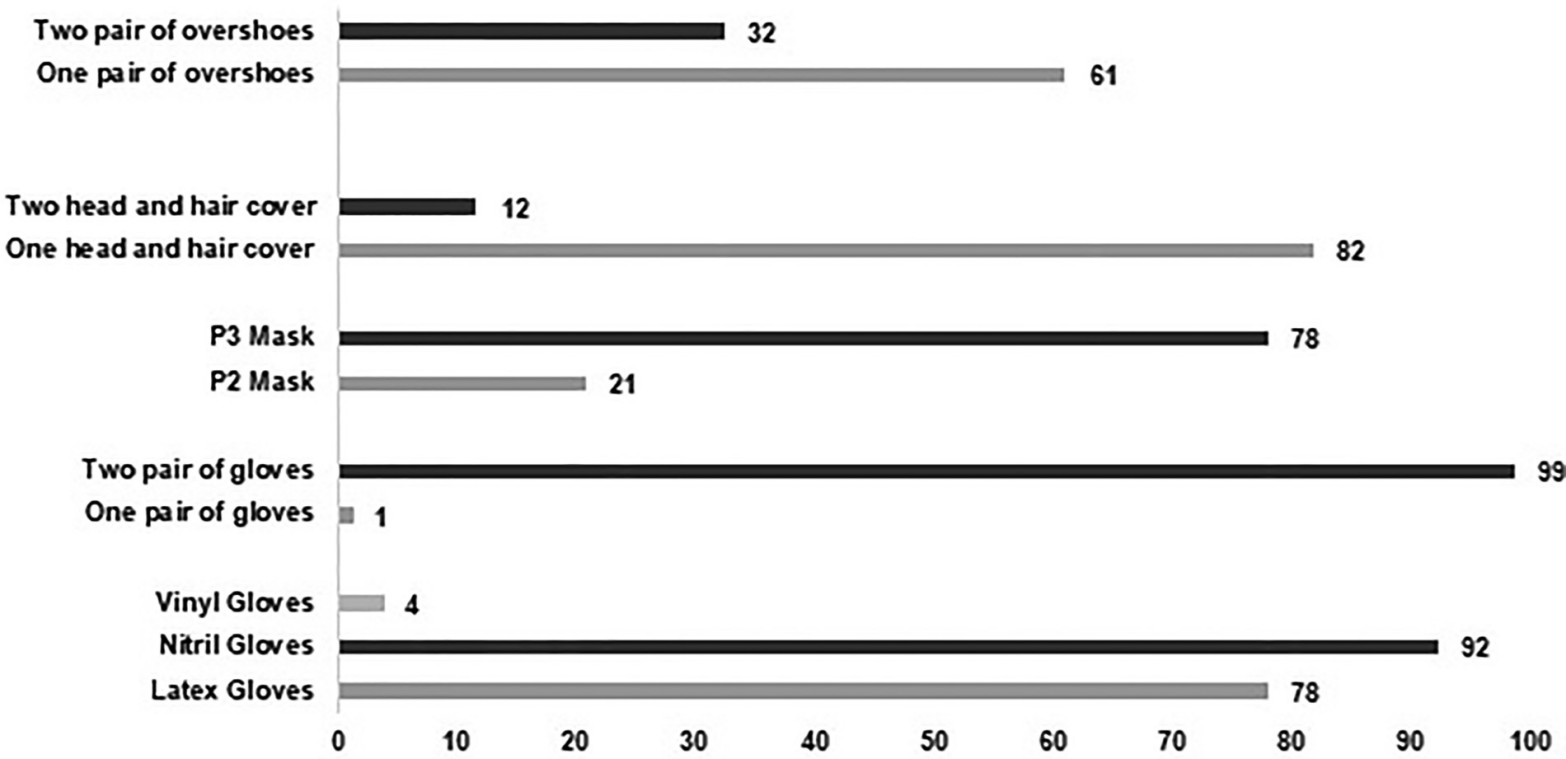
Rates of use of overshoes, head and hair covers, masks and gloves, according to particular characteristics.

Latex, nitrile, or neoprene gloves may be used for the manipulation of cytotoxic drugs if they have been validated for that purpose.^36^ All participants (*n* = 77) reported using gloves and gowns during cytotoxics manipulation (Table 1). A percentage of 92% of pharmacy technicians used nitrile gloves (*n* = 71), 78% used latex gloves and most of them (73%, *n* = 56), used both latex and nitrile gloves. Although described by a small percentage of professionals (4%; *n* = 3) the use of vinyl gloves should be discouraged (Figure 2).

**Table 1.** Rates and absolute frequencies of regular use of personnel protective equipment by pharmacy technicians (*n* = 77), during the manipulation of cytotoxic drugs.

Gloves	Gown	Mask	Head Cover	Overshoes	Scrubs	Specific Shoes	Protective Goggles
100% (*n* = 77)	100% (*n* = 77)	99% (*n* = 76)	94% (*n* = 72)	94% (*n* = 72)	95% (*n* = 73)	82% (*n* = 63)	25% (*n* = 19)

The self-reported adherence to gown and glove use in this study is among the highest ever described. In the various previously published papers, glove and gown use during reconstitution and preparation of cytotoxic drugs ranged between 49% and 99% for gloves and between 3% and 91% for gowns (respective gloves and gown adherence: 49% and 3% (*n* = 33),^22^ 76% and 36% (*n* = 632),^24^ 92% and 63% (*n* = 1932),^23^ 90% and 44% (*n* = 83),^25^ 91% and 21% (*n* = 824),^26^ 99% and 53% (*n* = 263),^27^ 95% and 85% (*n* = 88),^28^ 98% and 91% (*n* = 222)^29^ and 90% and 80% (*n* = 183)).^34^

Double-gloving was described by 99% of the participants (*n* = 76) (Figure 2), complying with the recommendations of the American Society of Health-System Pharmacists,^42^ which highlights the fact that wearing double gloves provides an additional barrier to possible contamination transfer as the hands are covered until the last item of PPE is removed. The use of two pairs of gloves when preparing cytotoxic drugs has been variable among different studies, with studies showing that all participants used two pairs of gloves,^20^ studies in which about 50% of the professionals used two pairs of gloves (*n* = 183),^34^ and studies in which the practice proved to be very rare or non-existent.^10^

Regarding the use of masks, 76 pharmacy technicians (99%) reported the use of either a P2 mask (*n* = 16; 21%) or a P3 mask (*n* = 60; 78%), both described as adequate by the ISOPP guidelines.^36^ Previous studies reported lower adherence to the use of masks at 47% (*n* = 222).^29^

Both head and hair covers and overshoes were used by 72 pharmacy technicians (94%). According to USP,^43^ these items protect from contact with hazardous drug residues and should be used. The use of a second shoe cover to be removed when exiting the hazardous drug compounding area is recommended, thereby limiting the contamination of other areas adjacent to the cleanroom.^36,43^ Only 25 pharmacy technicians (32%) included in this survey usually used double shoe covers.

### Practices and attitudes towards manipulation of cytotoxic drugs

**Figure 3.**
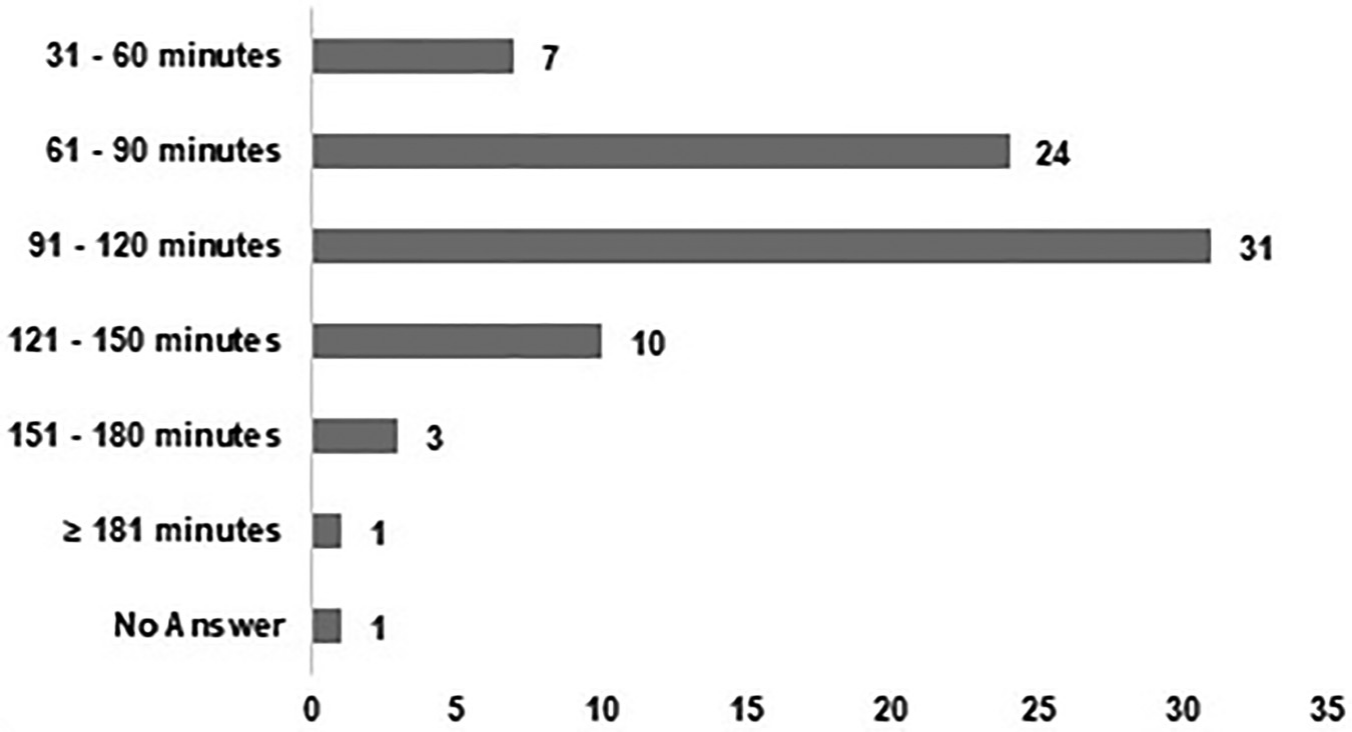
Time of consecutive manipulation of cytotoxic drugs without a break.

To maintain the concentration of the staff involved in the preparation of cytotoxic drugs, it is recommended that no more than 120 min be spent working at the BSC or isolator, without a break.^36^ Although most of the participants of the survey reported spending 120 min or less working without a break (*n* = 62, 81%), 18% of the pharmacy technicians (*n* = 14), admitted exceeding this time (Figure 3).

Several references recommend that sterile gauze should be placed around syringe connection sites.^29,37,44^ In the present study, most participants admitted to always complying with this recommendation (*n* = 45, 58%), but one participant admitted that he never did it and 15 (19%) admitted that they rarely did it (Table 2). The use of sterile gauze around syringe connection sites might be crucial to absorb spills and leakages, thus diminishing exposure. The simulation of cytotoxic drug manipulation with fluorescence substances such as quinine hydrochloride suggested that syringe connection sites are areas particularly prone to spills, evidencing the importance of placing a sterile gauze around syringe connections.^45,46^

**Table 2.** Rates and absolute frequencies of use of sterile gauze around syringe connection sites and performance of double checking by another professional during withdrawing and injecting drugs.

Use of sterile gauze around syringe connection sites				Double-checking during withdrawing and injecting drugs			
*1 (Never)*	*2 (Rarely)*	*3 (Often)*	*4 (Every time)*	*1 (Never)*	*2 (Rarely)*	*3 (Often)*	*4 (Every time)*
1% (*n* = 1)	19% (*n* = 15)	21% (*n* = 16)	58% (*n* = 45)	5% (*n* = 4)	3% (*n* = 2)	6% (*n* = 5)	86% (*n* = 66)

ISOPP states that double-checking during withdrawing and injecting drugs should be implemented. In the present study, most of the participants stated that the drugs that they prepared are always double-checked (*n* = 66, 86%). Still, four pharmacy technicians (5%) reported that theirs never are (Table 2). Training programs should highlight the benefits of increasing the accuracy of the preparations by systematically adopting the double-checking methodology.

**Figure 4.**
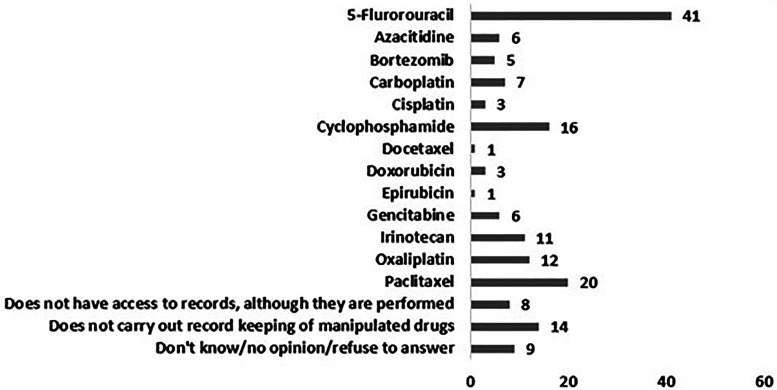
Drugs most manipulated by pharmacy technicians according to total mass recorded in the file of the manipulated drugs by each professional.

Participants were asked to indicate the three drugs they handled the most (in terms of total mass), according to their records of manipulation. Paclitaxel, cyclophosphamide and 5-fluorouracil were the three most manipulated drugs and 14 pharmacy technicians (18%) acknowledged that, unlike recommended,^38^ they did not perform these records. Eight of the professionals (10%) did not have access to the records of manipulated drugs, although they stated that they were performed (Figure 4).

### Engineering controls

**Figure 5.**
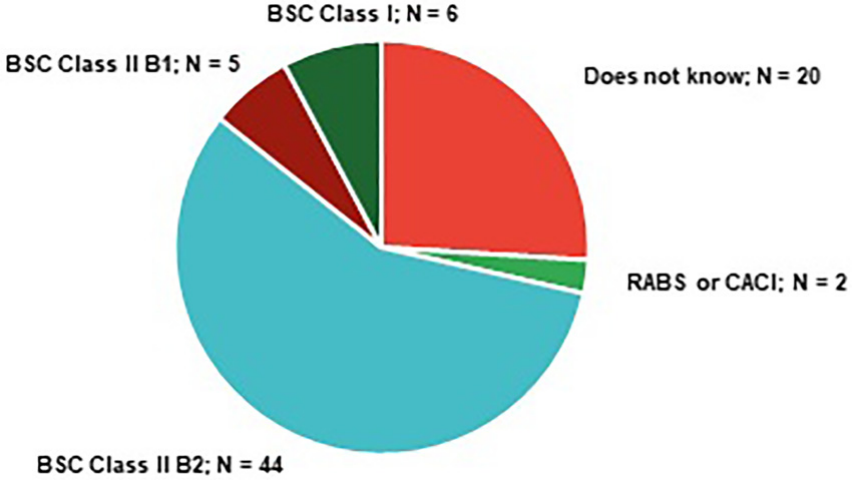
Containment primary engineering controls (C-PEC) used by pharmacy technicians in their hospital units (CACI = compounding aseptic containment isolator; RABS = restricted access barrier systems).

Regarding containment primary engineering controls (C-PECs) used in the manipulation of cytotoxics, 46 participants reported using C-PECs recognized by guidelines as adequate for manipulating all cytotoxic drugs^36^ – class II B2 BSC was pointed out by 44 participants (57%) and negative pressure restricted access barrier systems (RABS) or compounding aseptic containment isolator (CACI) was used by two participants (3%). Six participants (8%) reported using a Class I BSC, which must not be used for sterile products, and five participants (6%), chose the option “Class II Type B1” which is only suitable for minimal quantities of volatile cytotoxic drugs.^36^ Twenty pharmacy technicians, corresponding to 26% of the surveyed professionals, admitted that they did not know the classification of the C-PEC where they worked or had recently worked (Figure 5). Acknowledging the classification of the C-PEC, its basic functioning and features are essential to safely manipulate cytotoxic drugs. Accordingly, ISOPP states that the “Theory of containment devices and barriers” is one of the elements that should be contained in training programs for professionals working with those devices.

Several studies have demonstrated the effectiveness of closed-system transfer devices (CSTD) in reducing surface contamination.^47–52^ The few existing publications on adherence to the use of CSTD in cytotoxic handling reveal that they have not yet been adopted on a recurrent basis. In a study including 120 Canadian healthcare professionals who contacted cytotoxics in different functions (including in preparation), none of the participants used CSTD.^33^ Boiano, Steege^21^ revealed rates of “frequent use” of CSTD of 45%, while “never use” was answered by 47% of the respondents and “occasional use” gathered 8% of the responses (*n* = 1895). The main justifications presented for the low adherence to CSTD are the cost of the devices and the lack of training in their use. On the other hand, the use of withdrawal spikes, which are needleless systems, seems to occur more regularly (with 90% of the participants stating that they “Always” used them) (*n* = 1925).^21^ As with CSTD, spikes decrease the risk of accidental punctures and help to balance the pressure inside the vials.^21,37^ The main difference is that, unlike common withdrawal spikes, CSTD also prevent the escape of hazardous drug or vapor concentrations outside the system, enhancing the prevention of exposure.^36^ Nevertheless, drugs and device specifications should be carefully read before using these alternatives to needles, since some drugs preclude the use of some devices in their summary of product characteristics (SmPC). In the present study, none of the participants used CSTD. On the other hand, all participants admitted to using spikes, to some extent. Still, their criterion for using spikes seems to be, in some cases, unclear. Three participants (4%) assumed using spikes regardless of drug characteristics described in SmPC and 38 participants (49%) reported using spikes whenever recommended by the SmPC. However, when analyzing the SmPC of all cytotoxic drugs commercialized in Portugal in January of 2023, it was possible to verify that none of the SmPC recommends the use of spike. These results suggest that the use of the spike, by some pharmacy technicians, may not be exactly judicious. The remaining 36 pharmacy technicians (47%), have provided an acceptable justification, stating that they “used spike unless contra-indicated in SmPC”.

### Major concerns and difficulties of pharmacy technicians that manipulate cytotoxics

Pharmacy technicians that participated in this study had the opportunity to express their major concerns and difficulties when manipulating cytotoxics, in an open-ended question. Twenty-nine participants shared a total of 41 concerns and difficulties that could be categorized into nine different groups. Excessive workload (*n* = 7) and insufficient human resources (*n* = 7) were the most frequent answers, followed by insufficient training (*n* = 6) and future health consequences of the performed tasks (exposure to cytotoxic drugs and routine movements) (*n* = 6) (Table 3). These results further emphasize the need for the education and training of more professionals that are conveniently prepared to manipulate cytotoxic drugs, adopt safer procedures, and enable a higher rotation of professionals.

**Table 3.** Concerns and/or difficulties expressed by pharmacy technicians that manipulate cytotoxic drugs.

Concern/Difficulty	*n*
Excessive workload	7
Insufficient human resources	7
Insufficient training	6
Future health consequences of the performed tasks	6
Inadequate physical conditions	5
Technical difficulties during the manipulation itself/task demand	4
Lack of material or poor quality of material	3
Lack of clinical monitoring	2
Lack of standardisation of recommendations	1

## Conclusion

In the present survey, it was possible to verify that Portuguese pharmacy technicians generally receive training before their admission in the manipulation of cytotoxic drugs. It was also possible to testify the generic overall adoption use of most critical PPE items – gloves (in duplicate), gown, and mask. In fact, regarding both prior training and PPE use, the surveyed professionals have demonstrated higher levels of adherence to recommendations than most of the previously studied populations. However, the participation of the employing institutions in regular training and assessment of their professionals, after admission in manipulating cytotoxic drugs, is frequently unsatisfactory. Training programs should encourage more frequent use of sterile gauze around syringe connections, double checking procedures of the preparations, maximum time of manipulation without a break of 120 min and a more profound knowledge, with subsequent more judicious use of engineering controls. The concerns of pharmacy technicians should not be disregarded. Accordingly, the implementation of more and more frequent education and training programs to better prepare more pharmacy technicians in manipulating cytotoxic drugs might contribute to a decrease in the reported excessive workload.

FM conceived the study. DC, IS, MR, PC, and FM were involved in gaining ethical approval. DC, IS and MR developed the web-based survey. FM corrected the web-based survey according to experts’ opinions and following the pilot-test application. DC, IS, and MR disseminated the questionnaire. DC, IS, MR, PC, and FM analyzed the results. FM wrote the first draft of the manuscript. All authors reviewed and edited the manuscript and approved the final version of the manuscript.
